# 

**Published:** 1884-06

**Authors:** 


### JUNE, 1884.

### CONTENTS.

ORIGINAL PAPERS:— *Page.*

The Nature and Treatment of Chorea. By E. LONG FOX, M.D., F.R.C.P. ... 73

Ingrowing Toe-nail. By THE EDITOR ... 99

CLINICAL RECORDS:—

Short Reports of Twelve Cases of Ligature of some of the Main Arteries occurring during the past few months at the Bristol Royal Infirmary. By J. PAUL BUSH ... 110

Case of Cerebral Abscess following Slight Injury. By A. N. GODBY GIBBS ... 121

Case of Malignant Pustule. By LIONEL A. WEATHERLY, M.D. ... 124

Case of Tumour (Columnar Papilloma) in Fourth Ventricle of Brain, with Hydrocephalus. By J. TAYLOR, M.R.C.S. ... 128

NOTES ON BOOKS ... 132

MEDICAL EXTRACTS ... 136

